# LINC00858 stabilizes RAN expression and promotes metastasis of gastric cancer

**DOI:** 10.1186/s13062-022-00355-5

**Published:** 2022-12-17

**Authors:** Yunxin Lu, Qi Meng, Long Bai, Ruobing Wang, Yong Sun, Jiaqi Li, Jun Fan, Tian Tian

**Affiliations:** 1 State Key Laboratory of Oncology in South China, Collaborative Innovation Center for Cancer Medicine, Sun Yat-Sen University Cancer Center, Guangzhou, 510060 China; 2 Department of Medical Biochemistry and Molecular Biology, School of Medicine, Jinan University, Guangzhou, 510632 China

**Keywords:** LINC00858, Gastric cancer, Metastasis, YY1, RAN

## Abstract

Metastasis constitutes one of the major causes of tumor-related death in gastric cancer (GC), and understanding key events in the initiation of this phenotypic switch may provide therapeutic opportunities. Long noncoding RNAs (lncRNAs) are emerging as molecules that play vital roles in tumorigenesis and metastasis. In this study, we aimed to identify metastasis-related lncRNAs in the context of GC. The lncRNAs overexpressed in tumor tissues and positively associated with overall survival were screened out using the TCGA database. qPCR assays in clinical samples showed that LINC00858 was significantly upregulated in GC tissues compared with normal counterparts. Functional analysis suggested that LINC00858 depletion attenuated the migration, and invasion of cancer cells in vitro and suppressed the metastasis of xenografted tumors in vivo. Mechanistically, LINC00858 could interact with the metastasis-associated RAN and stabilize its protein expression by decreasing posttranslational ubiquitination. The transcription factor YY1 could bind to the promoter of LINC00858 to upregulate its expression in GC cells. Moreover, overexpression of YY1 and RAN was positively associated with upregulation of LINC00858 in GC tissues. Our results suggest that LINC00858 might play a role in GC metastasis, and be a diagnostic biomarker and potential therapeutic target.

**Supplementary Information:**

The online version contains supplementary material available at 10.1186/s13062-022-00355-5.

## Introduction

Gastric cancer (GC) is one of the most common malignant tumors worldwide. The incidence of GC ranks fifth, and its mortality ranks third globally [1]. Surgery plus adjuvant chemotherapy provides a median overall survival (OS) of 50 months for locally advanced or lymph node positive patients [2]. However, the prognosis of metastatic GC (mGC) is extremely poor, with a median OS of less than 15 months, even in the era of immunotherapy [3], generating an urgent need to identify novel diagnostic markers and to elucidate key mechanisms in the pathogenesis of mGC [4].

Metastasis has been identified as one of the major causes of death in patients diagnosed with GC [5]. Theoretically, tumor metastasis is a multistep process that involves both genetic and epigenetic alterations [6]. As newly discovered epigenetic regulators, long noncoding RNAs (lncRNAs) have become hot topics in cancer research in recent years [7], and aberrant expression or dysfunction of lncRNAs is closely related to GC development and metastasis [8, 9]. For example, Sun TT et al. reported that GClnc1 affects proliferation, invasiveness, and metastasis by acting as a scaffold to recruit the WDR5 and KAT2A complex and modify the transcription of target genes in GC [8]. Overexpression of GMAN is associated with GC metastasis by promoting translation of ephrin A1 through competitive binding with GMAN-AS [9]. Thus, an in-depth study of the molecular mechanisms of lncRNAs, especially metastasis-related ones, will provide new strategies for the diagnosis and individual treatment of GC.

RAN is a Ras-related GTPase that is involved in nuclear-cytoplasmic transport, cell cycle regulation, and cell transformation [10]. Moreover, RAN is overexpressed in multiple tumors, including breast, renal and gastric cancer, and is associated with tumor progression, metastasis and poor patient survival [11–14]. Zaoui K et al. [12] showed that the binding of RAN with RhoA and protection from ubiquitination were essential for the activation of downstream metastatic signaling and the subsequent invasive progression of ovarian cancer. Interactions with other proteins have also been reported to be vital in the prometastatic roles of RAN [11]. However, no reports have investigated the interaction of lncRNAs with RAN, and little is known about how the expression and functions of RAN are regulated in GC.

In this study, through bioinformatic analysis, LINC00858 was found to be overexpressed in GC and to be associated with poor overall survival of patients, which was further validated using clinical samples collected at our institution. Furthermore, knockdown of LINC00858 significantly reduced GC cell migration, invasion, and metastasis of xenografted tumors in vitro and in vivo. Mechanistically, LINC00858 promoted GC metastasis by directly interacting with the metastasis-associated RAN and stabilizing its protein expression by decreasing posttranslational ubiquitination. Additionally, the transcription factor YY1 could bind to the promoter of LINC00858 to upregulate its expression. Taken together, our study identified a metastasis-associated LINC00858 in GC through in silico analysis and elucidated its prometastatic roles and potential mechanisms via experimental assays.

## Materials and methods

### Cell lines and cell culture

The gastric cells used included GES1, AGS and SGC7901 cells from ATCC and SNU216, BGC823, HGC27, MGC803, NUGC4, MKN45 and MKN74 cells from the Institute of Basic Medical Sciences of the Chinese Academy of Medical Sciences. RPMI-1640 or DMEM (Invitrogen) with 10% FBS (HyClone) was used for cell culture in a humidified cell incubator at 37 °C with an atmosphere of 5% CO_2_. Only cell lines not passaged for more than one month after resuscitation were processed for in vitro or in vivo assays in our study. All cells tested negative for mycoplasma and were authenticated by short tandem repeat DNA fingerprinting at the Medicine Lab of the Forensic Medicine Department of Sun Yat-sen University (Guangzhou, China).

### Human tissue specimens

Samples from 228 GC patients who underwent radical surgery at Sun Yat-sen University Cancer Center (SYSUCC, Guangzhou, China) were collected, among which 55 paired fresh normal gastric tissues were archived, and 55 GC samples suitable for immunohistochemistry assays were paraffin embedded. In addition, 100 paired colorectal cancer and adjacent normal tissues were used in this study. No neoadjuvant radiotherapy or chemotherapy was allowed in both cohorts. All cases were pathologically diagnosed as gastric cancer or colorectal cancer and staged according to the TNM staging system of the American Joint Committee on Cancer (AJCC 7th ed., 2010). All samples were immediately frozen in liquid nitrogen after resection and stored at − 80 °C for RNA extraction. Moreover, another cohort containing 346 formalin-fixed, paraffin embedded tissue samples collected from GC patients who underwent surgery at Sun Yat-sen University Cancer Center (Guangzhou, China) were retrieved. Informed consent and approval of use of cancer tissues in our study were obtained from all patients and the Committee for Ethical Review of Research involving Human Subjects of Sun Yat-sen University, respectively. All clinicopathologic characteristics of the included GC patients, whose samples were used for detection of LINC00858 expression, are listed in Additional file 1: Table S1.

### RNA extraction, subcellular fractionation and quantitative polymerase chain reaction (qPCR) analysis

TRIzol reagent (Invitrogen) was used for RNA extraction from fresh samples or cell lines. RNA was reversed transcribed to cDNA using a Prime Script RT Master Mix Kit (Takara), and then the cDNA was used as a template to perform qPCR with SYBR Green Mix (Promega) according to the manufacturer's instructions and then subjected to the ABI 7500 Fast Real-Time PCR System (Applied Biosystems). Cytoplasmic and nuclear RNA were separated using a Cytoplasmic & Nuclear RNA Purification Kit (Norgen) according to the manufacturer’s instructions and then subjected to qPCR analysis. The specific primers sequences used in our study are shown in Additional file 1: Table S4. Each sample was run in triplicate, and fold changes were calculated using the relative quantification 2^−△△CT^ or the △CT method.

### Small interfering RNAs (siRNAs), vectors, cell transfection, and lentivirus

The siRNAs targeting YY1 were synthesized by RiboBio (Guangzhou, China). Expression vectors of full-length LINC00858 and RAN were purchased from OBIO Technology (Shanghai, China). The expression vectors for 3FLAG-tagged MS2 coat protein (MCP) and MS2-tagged LINC00858 were provided by OBIO Technology (Shanghai, China). All constructs were verified by sequencing. Cell transfection was performed using Lipofectamine 3000 reagent (Invitrogen) or RNAiMAX Reagent (Invitrogen). The cells were harvested for assays 48 h after transfection. HGC27 and MKN74 cells were transfected with lentiviruses containing LINC00858 short hairpin RNA (shRNA) (OBIO, China), and stable cell lines were selected by treatment with 2 μg/mL puromycin for one week.

### Transwell migration and invasion assays and cell proliferation analysis.

Transwell chambers (Corning, USA) with a membrane pore size of 8 μm were coated without or with Matrigel (BD Biosciences) and used for migration or invasion assays, respectively. Briefly, cells trypsinized and suspended in 100 μl serum-free medium were plated in the upper chamber, while the medium supplemented with 20% FBS was added into the lower chambers as the chemoattractant. After incubation for different period, the non-migrated cells on the top surface were swabbed off gently and the cells attached to the bottom of the filters were fixed with methanol, stained with 0.1% crystal violet. The number of migrated or invaded cells was counted using an inverted microscope. The number of cells seeded in the upper chamber and culturing period were provided in Additional file 1: Table S5. Cell proliferation was measured with MTS (Qiagen, Hilden, German) according to the manufacturer’s instructions.

### Protein extraction, immunoblotting and immunohistochemical analysis

RIPA lysis buffer (Cell Signaling Technology) containing PMSF (Cell Signaling) was used for protein extraction from cells and fresh samples. The protein concentrations were measured using a BCA Protein Assay Kit (Thermo Fisher Scientific), and equal amounts were separated by sodium dodecyl sulfate–polyacrylamide gel electrophoresis and transferred to polyvinylidene difluoride membranes. Primary antibodies including RAN (Abcam, ab233762), YY1(CST, 46395S), β-Actin (Sigma, A5441) and HSP90 (CST, 4874) were used to detect the expression of individual proteins. Proteins were visualized with Western Lightning Chemiluminescence Reagent Plus (PerkinElmer, Waltham). The MG132 and Cycloheximide (CHX) were purchase from Selleck Chemicals (Houston, TX, USA) and MedChem Express (Shanghai, China), respectively. The final concentration of MG132 and CHX used for immunoblotting assays were 10 μM and 20 μg/ml. The immunohistochemical analysis was conducted according to our previous reports [15, 16] using antibodies against RAN (Abcam, ab233762), YY1(CST, 46395S).

### In vitro transcription and RNA pull down assay

A vector of LINC00858 containing a T7 promoter used in in vitro transcription assays was synthesized by RiboBio (Guangzhou, China). The MEGAscript™ T7 Transcription Kit (Invitrogen) was used for the in vitro transcription assay according to the manufacturer’s instructions. Then, the RNA was biotinylated using a Pierce RNA 3' End Desthiobiotinylation Kit (Thermo Scientific) according to the manufacturer’s instructions. An RNA pulldown assay was performed using a Pierce Magnetic RNA–Protein Pull-Down Kit (Thermo Scientific). Briefly, biotinylated RNA was conjugated with streptavidin magnetic beads and then incubated with cell lysates or purified RAN protein at 4 °C for 4 h before washing and elution of RNA-binding protein complexes. Eluted proteins were subjected to mass spectrometry (MS) or immunoblotting analysis.

### RIP and chromatin immunoprecipitation (ChIP) assays

A Magna RNA-binding protein immunoprecipitation kit (Millipore) was used for the RIP assay according to the manufacturer’s instructions. Briefly, negative control rabbit IgG, human anti-RAN antibody (Abcam, ab155103) or anti-Flag tag antibody (CST, 8146) was incubated with magnetic beads at 37 °C for 30 min. Then, cell lysates were incubated with magnetic bead-antibody complexes at 4 °C overnight. After proteinase K digestion, RNAs were extracted, purified, reverse transcribed to cDNA, and subjected to qPCR. The RNA levels were normalized to the input (10%) in each group. The Millipore ChIP kit (17-10085) was used to carry out ChIP assay according to the manufacturer’s instructions. DNA fragments bound to YY1 were subjected to qPCR analysis using specific primers (Additional file 1: Table S4). Anti-YY1 (CST, 46395S) was used in ChIP assays. Detailed protocols were reported in a previous report [17].

### Xenograft assays

For the lung metastasis model, 5 × 10^6^ HGC27 cells stably expressing sh-LINC00858 or sh-Ctrl were injected into the tail veins of nude mice. At the indicated time points, lung metastasis was monitored using a Xenogen IVIS 100 bioluminescent imaging system according to the manufacturer’s instructions after intraperitoneal injection of D-luciferin (Goldbio, cat. no. LUCK-1). Two months later, the mice were sacrificed, and the lungs were fixed and paraffin embedded for hematoxylin and eosin (H&E) staining. For the in-situ metastasis model, 1 × 10^6^ HGC27 cells stably expressing sh-LINC00858 or sh-Ctrl were injected subcutaneously. The formed tumors were equally divided into several pieces of approximately 1mm^3^ and transplanted into the stomach of nude mice. A month later, mice were sacrificed, and hepatic and lymph node metastases were photographed and subjected to pathological analysis. Our animal study was approved by the Institutional Animal Care and Use Committee of Sun Yat-Sen University.

### Statistical analysis

All experiments were repeated at least three times. The data are presented as the mean ± SD unless specifically indicated and were analyzed using SPSS (version 20.0) or GraphPad Prism 8.0. Student’s t test, one-way ANOVA or chi-square test was used as appropriate. Survival curves were generated using Kaplan–Meier method and compared using the log-rank tests. *P* values less than 0.05 were considered statistically significant.

## Results

### Overexpression of LINC00858 in GC was identified in the TCGA database and GC cohort.

**Fig. 1:**
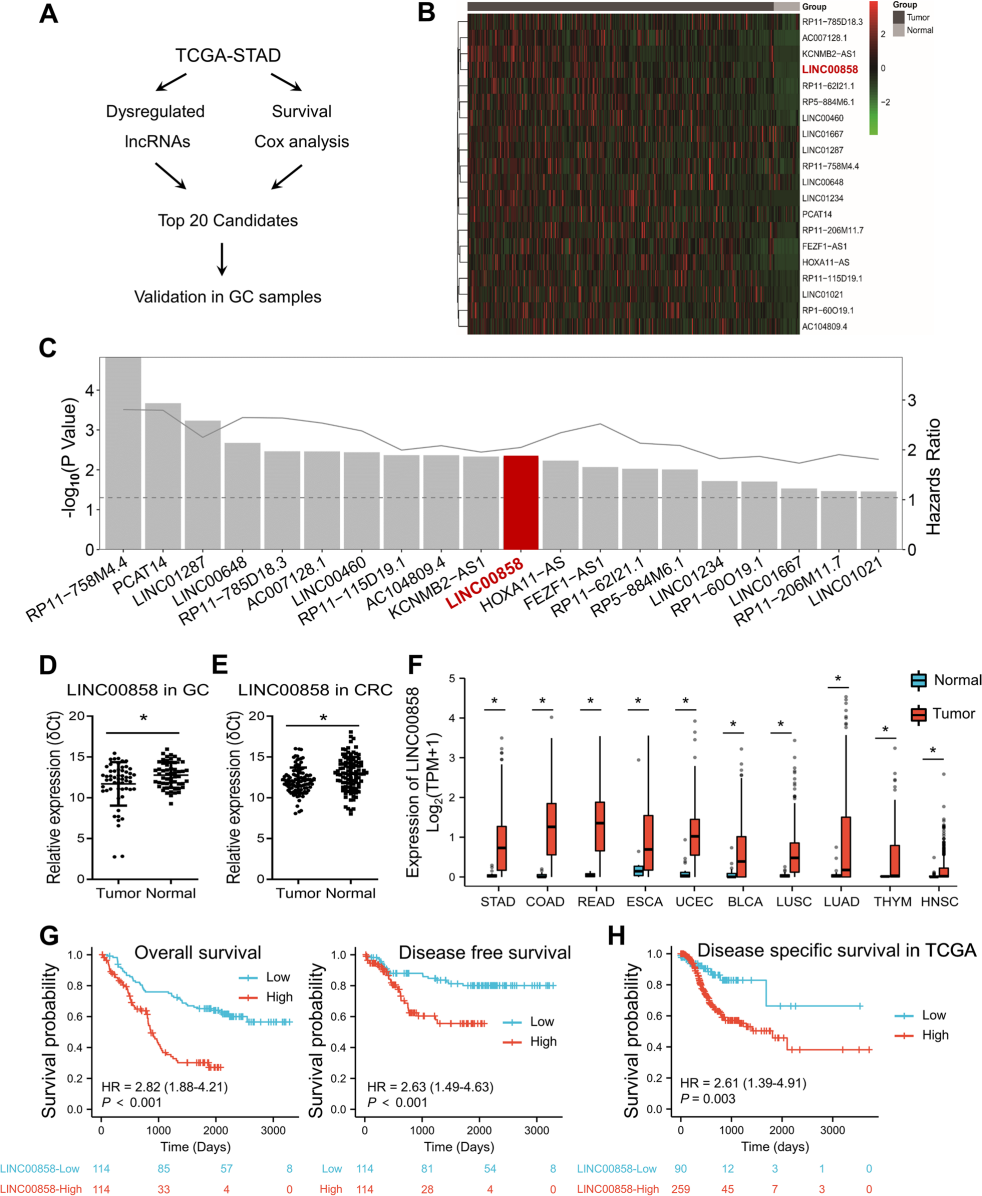
LINC00858 is overexpressed in TCGA-STAD database and is associated with poor clinical outcome. **A** Flowchart of this study. **B** Heatmap of the top 20 candidate lncRNAs that were highly expressed between STAD and normal gastric tissues and significantly associated with OS in the TCGA database. **C** Correlations between the 20 lncRNAs with prognosis in STAD according to the TCGA database. The column represents *P* value of each lncRNA and the dotted line represents *P* = 0.05. The solid line represents the HR (Hazards Ratio) value. **D** qPCR detection of LINC00858 expression in 55 paired tumor and normal gastric tissues. **E** qPCR detection of LINC00858 expression in 100 paired tumor and normal colorectal tissues. Data in D and E are shown as the mean ± SD. △CT on Y-axis indicates the Ct value of the internal control subtracted from that of the target genes, and lower values indicate higher expression. **F** LINC00858 expression in TCGA tumors and normal tissues with the data of the GTEx database as controls. **G** Kaplan‒Meier analysis of the correlation between LINC00858 expression and overall survival and disease free survival in the SYSUCC cohort. **H** Kaplan–Meier analysis of the correlation between LINC00858 expression and disease specific survival in the TCGA-STAD database. The *P* values in D-F were calculated using a two-sided paired or unpaired Student’s t test. *P < 0.05

To identify lncRNAs involved in GC progression, we first mined GC RNA-seq data from the online TCGA dataset (TCGA-STAD) (Fig. 1A). The upregulated lncRNAs (fold change ≥ 2 and *P* value < 0.05) in GC samples compared with normal gastric tissues were screened out. On the other hand, lncRNAs significantly associated with OS were identified via Cox analysis (*P* < 0.05). Overlapped candidates were ranked according to the fold change and the top 20 are shown in a heatmap (Fig. 1B), while the *P* values and HRs of these top 20 upregulated lncRNAs in the survival analysis are presented in Fig. 1C. Previous reports by other groups have showed that LINC00460 [18], LINC01021 [19] and LINC01234 [20] play oncogenic roles in GC, confirming the reliability of our analysis (Fig. 1B and C), and these lncRNAs were therefore excluded from further investigation.

Then we performed qPCR assays to examine their expression in paired tumor and normal tissues of GC (n = 55) and colorectal cancer (CRC, n = 100) enrolled in our institute. The expression of LINC00858 was significantly upregulated in tumor tissues compared with normal tissues in both GC (Fig. 1D) and CRC (Fig. 1E) cohort and was therefore selected for further analysis. Furthermore, for TCGA tumors with the GTEx database as controls, LINC00858 was significantly upregulated in ten cancer types including GC (Fig. 1F) and further validated in paired tumor and adjacent normal tissues from TCGA (Additional file 1: Fig. S1A). These results indicated that LINC00858 overexpression is a common phenomenon across multiple tumor types, especially in gastrointestinal malignancies.

To investigate the clinical relevance of LINC00858 in GC, all patients were divided into high expression and low expression groups according to the qPCR results using median value as cutoff point. Statistical analysis revealed that high expression of LINC00858 was significant correlated with age, lymph node status, and TNM stage but not with other clinical parameters (Additional file 1: Table S1). Furthermore, Kaplan–Meier survival analysis showed that high expression of LINC00858 was related to poor overall survival and disease free survival (Fig. 1G). Multivariate analysis revealed that differentiation state, infiltration of peritumoral tissues, TNM stage, and LINC00858 expression were all independent prognostic factors in gastric cancer (Additional file 1: Table S2). In addition, upregulated expression of LINC00858 was found to be associated with poor disease specific survival (Fig. 1H), overall survival and progress free interval (Additional file 1: Fig. S1B) in the TCGA-STAD database, which was further validated in the TCGA-COAD cohort (Additional file 1: Fig. S1C). Taken together, LINC00858 was identified to be overexpressed in GC and positively associated with poor survival.

### Overexpression of LINC00858 promotes GC cell migration and metastasis.

**Fig. 2:**
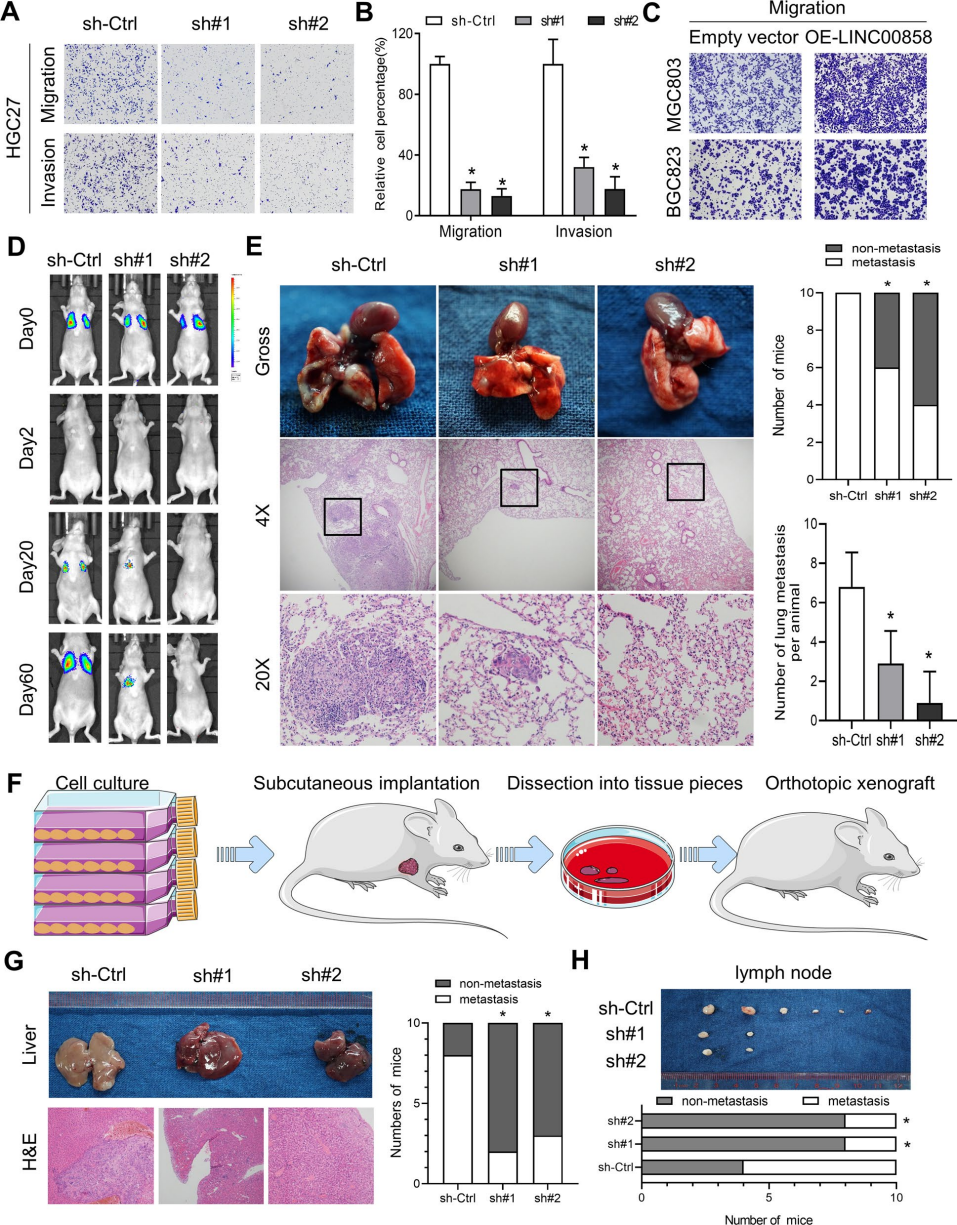
LINC00858 is overexpressed in multiple GC cell lines and promotes cell migration and invasion. **A** Representative images of migration and invasion analysis in HGC27 cells upon knockdown of LINC00858. **B** Quantification of migrated and invaded HGC27 cells upon knockdown of LINC00858. **C** Representative images of migrated MGC803 and BGC823 cells upon overexpression of LINC00858. **D** Representative luciferase imaging of lung metastatic cells in nude mice after LINC00858 knockdown. **E** Representative results of hematoxylin and eosin (HE) staining (left) of metastatic lung nodules from mice injected with LINC00858 knockdown and control HGC27 cells via the tail vein. Number of mice with or without lung metastases were analyzed (Upper right). Metastatic nodules under microscope were counted and recorded (Lower right). **F** Schematic diagram illustrating the generation of orthotopic xenografted models. **G** Representative liver metastasis of HGC27 cells inoculated orthotopically were photographed and stained with HE. The number of mice with or without liver metastasis was documented. **H** Representative metastatic lymph nodes of HGC27 cells inoculated orthotopically were photographed, and the number of mice with or without lymph node metastasis was documented. The *P* values in B and E (lower) were calculated using two-sided unpaired Student’s t test, E (upper) and those in G and H were calculated using chi-square test. *P < 0.05

To investigate the potential biological role of LINC00858 in gastric cancer progression, the expression of LINC00858 was first detected in gastric cancer cells and epithelial cells (GES1). As shown in Additional file 1: Fig. S2A, LINC00858 was overexpressed in cancer cells compared with GES1 cells. Second, we transfected HGC27 and MKN74 cells, which had relatively high basal expression of LINC00858, with two different shRNAs targeting LINC00858 (sh#1, sh#2) and control shRNAs (sh-Ctrl), and the knockdown efficiency was confirmed by qPCR (Additional file 1: Fig. S2B). The cell proliferation of HGC27 and MKN74 remained unchanged upon knockdown of LINC00858 (Additional file 1: Fig. S2C). Transwell assays showed that LINC00858 silencing significantly inhibited the migration and invasion of HGC27 and MKN74 cells (Fig. 2A, 2B and Additional file 1: Fig. S2D). On the other hand, overexpression of LINC00858 in MGC803, BGC823 and SGC7901 cells (Additional file 1: Fig. S2E) obviously enhanced cell migration and invasion (Fig. 2C and Additional file 1: Fig. S2F–H). Third, we isolated a primary gastric cancer cell line and explored the roles of LINC00858 (Additional file 1: Fig. S2I). Knockdown of LINC00858 significantly suppressed the migration and invasion of the primary GC cells (Additional file 1: Fig. S2J), which further validated the prometastatic roles of LINC00858.

To assess the effect of LINC00858 on tumor metastasis in vivo, HGC27 cells stably expressing sh-LINC00858 (sh#1, sh#2) or sh-Ctrl were transplanted into nude mice via the tail vein. Fluorescence imaging was used to monitor lung metastasis at the indicated times (Fig. 2D). The results showed that sh-LINC00858 cells formed remarkably smaller lung metastases than sh-Ctrl cells (Fig. 2E). Hematoxylin and eosin (H&E) staining of dissected lungs revealed significantly fewer metastatic nodes in the sh-LINC00858 group than in the sh-Ctrl group (Fig. 2E). Furthermore, in the gastric orthotopic tumor metastasis model (Fig. 2F), the sh-Ctrl gastric cancer cells exhibited significantly more frequent liver metastasis (Fig. 2G) and lymph node metastasis (Fig. 2H) compared with the knockdown groups. Altogether, these results suggested that LINC00858 may function as a metastasis related oncogenic lncRNA in GC both in vitro and in vivo.

### LINC00858 is directly associated with the metastasis-associated protein RAN

**Fig. 3:**
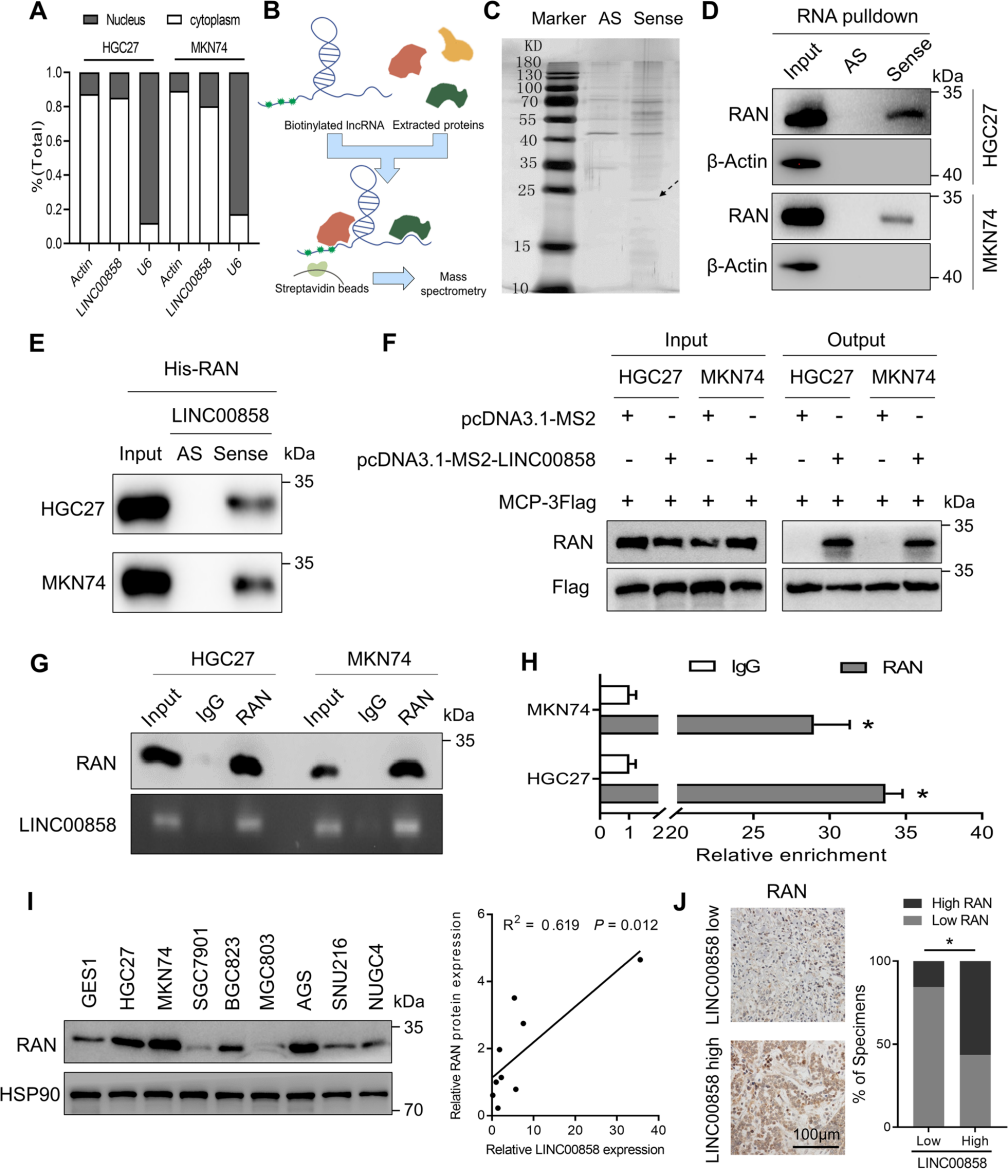
The metastasis-associated protein RAN was identified as the direct interacting protein of LINC00858 in GC. **A** Expression of LINC00858 in the cytoplasm and nucleus was detected via qPCR. **B** Diagram illustrating steps to identify interacting proteins. **C** Representative gels of immunoprecipitated proteins. **D** In vitro-synthesized LINC00858 was incubated with protein lysates from HGC27 and MKN74 cells. RAN protein was pulled down by biotin-labeled LINC00858 but not LINC00858 antisense RNA. **E** In vitro-synthesized LINC00858 was incubated with purified 6X His-tagged recombinant RAN. Purified RAN protein was pulled down by biotin-labeled LINC00858 but not LINC00858 antisense RNA. **F** Western blotting detection of RAN binding to LINC00858 after FLAG-MCP-MS2 pull-down assays. **G**, **H** RIP assays indicated that LINC00858 was precipitated with RAN in whole cell lysates of HGC27 and MKN74 cells (**G**), and the amount was measured by qPCR (**H**) and electrophoresis detection (**G**). **I** Western blotting of RAN expression in GES1, HGC27, MKN74, SGC7901, BGC823, MGC803, AGS, NUGC4 and SNU216 cells (left panel). Correlations between the mRNA expression of LINC00858 and the protein level of RAN in GC cells (right panel). **J** Representative images of immunohistochemical analysis of RAN in LINC00858 low- and high-expressing samples (left panel). Associations between LINC00858 expression and protein level of RAN in GC tissues (right panel). The *P* values in H were calculated using two-sided unpaired Student’s t test, while that in I (right) and J (right) was calculated using Pearson’s correlation analysis and chi-square test. *P < 0.05

Given that LINC00858 plays an important role in the metastasis of gastric cancer both in vitro and in vivo, we first detected the expression of LINC00858 via fractionation of cytoplasmic and nuclear RNA. As shown in Fig. 3A, LINC00858 was predominantly located in the cytoplasm. Moreover, RNA pull-down assays were performed to identify proteins interacting with LINC00858 in gastric cancer cells (Fig. 3B). Gel electrophoresis showed that several specific bands at ~ 24 kDa, 38 kDa and 60 kDa were enriched in the sense group in contrast with the antisense control (Fig. 3C). Proteins interacting with LINC00858 were identified via mass spectrometry, of which only three proteins, ARG1, RAN and HNRNPD were annotated to be related to tumor metastasis in the Human Cancer Metastasis Database (HCMDB), and according to the emPAI, RAN ranks first (Additional file 1: Table S3) and was therefore selected for following assays.

The interaction between LINC00858 (but not antisense LINC00858) and RAN was further confirmed by RNA pulldown following immunoblotting assays (Fig. 3D). Besides, in vitro purified protein RAN could also be pulled down by biotin-labelled LINC00858 but not LINC00858 anti-sense RNA (Fig. 3E). Direct interaction between LINC00858 and RAN was further validated using 3FLAG-tagged MS2 coat protein (MCP) and MS2-tagged LINC00858 (Fig. 3F). Moreover, RIP assays indicated that the RAN antibody could significantly enrich LINC00858, but the IgG antibody could not (Fig. 3G and H). In addition, immunoblotting of RAN in the cell lines showed that RAN was overexpressed in GC cells compared with GES1 cells and was positively associated with the mRNA level of LINC00858 (Fig. 3I). We then analyzed protein expression of RAN via IHC in 55 gastric cancer samples (Fig. 3J). Positive association between LINC00858 and RAN were shown in Fig. 3J, providing further evidence of their regulatory relation. Altogether, these results showed that LINC00858 could bind with RAN and regulate its protein expression in GC.

### LINC00858 protects RAN from ubiquitination and subsequent proteasomal degradation.

**Fig. 4:**
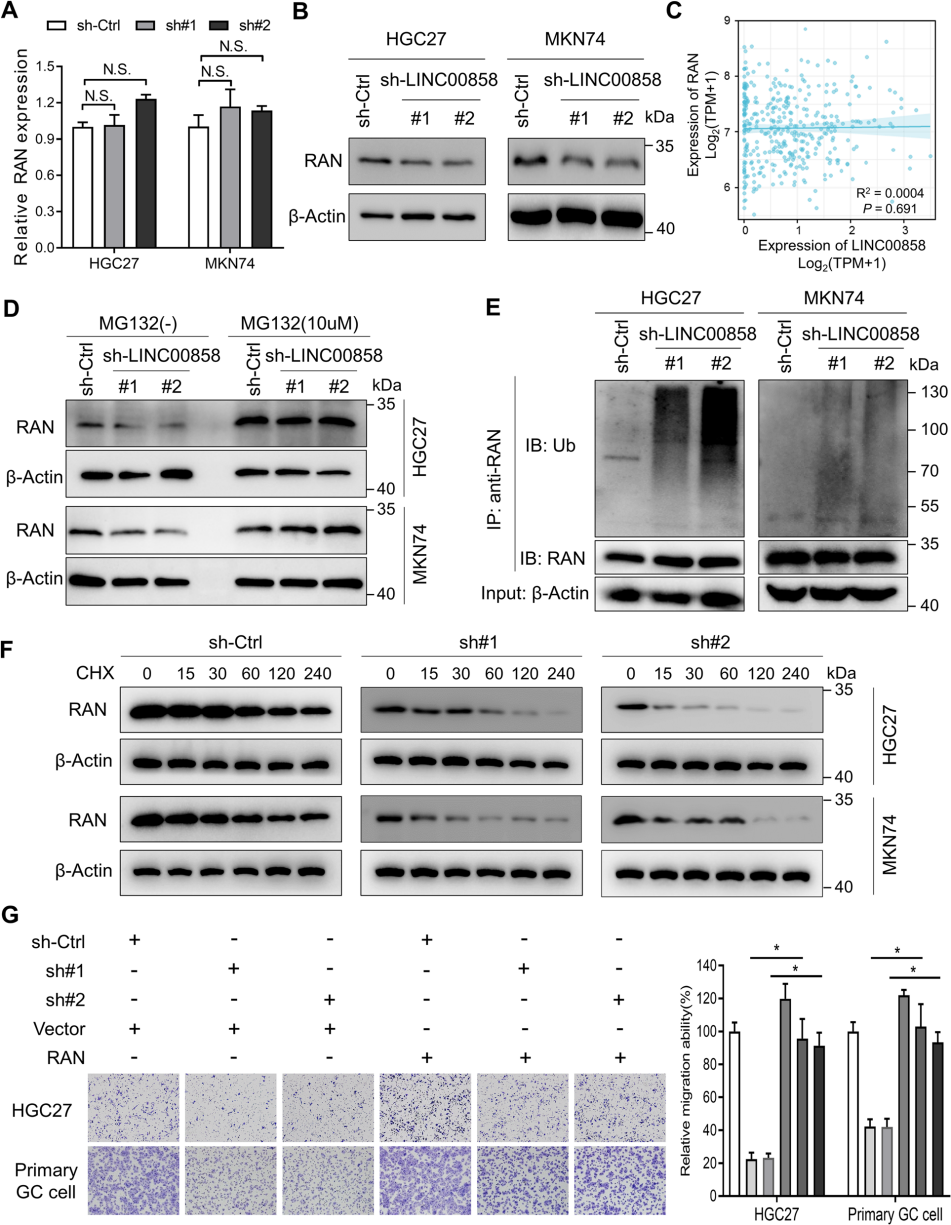
LINC00858 protects RAN from ubiquitination and proteasomal degradation in GC. **A** qPCR detection of RAN expression in HGC27 and MKN74 cells upon knockdown of LINC00858. **B** Western blotting of RAN expression in HGC27 and MKN74 cells upon knockdown of LINC00858. **C** Correlations between RAN and LINC00858 in the TCGA-STAD database. **D** Downregulation of RAN caused by LINC00858 knockdown was abolished by MG-132(10 μM, 12 h) in HGC27 and MKN74 cells. **E** IP assays showed that LINC00858 knockdown resulted in increased ubiquitination of RAN in HGC27 and MKN74 cells. **F** HCG27 and MKN74 cells with or without shRNAs specific for LINC00858 were treated with 20 μg/ml CHX for the indicated periods of time. RAN levels were analyzed by western blotting. **G** The effects of LINC00858 knockdown on migration and invasion were reversed by overexpression of RAN in HGC27 and primary GC cells. Representative images (left panel) and quantification data (right panel) are shown. The *P* values in A and G were calculated using two-sided unpaired Student’s t test. *P < 0.05 and N.S. indicate nonsignificant

To further elucidate the effects of LINC00858 binding to RAN, we performed qPCR and immunoblotting assays to detect the expression of RAN after LINC00858 knockdown. As shown in Fig. 4A and B, the protein level of RAN was significantly reduced after silence of LINC00858, although the mRNA level remained unchanged in HGC27 and MKN74 cells. Consistently, upregulation of LINC00858 resulted in overexpression of RAN in MGC803, BGC823 and SGC7901 cells (Additional file 1: Fig. S3A). This revealed that post-translational modifications may affect the stability of RAN in the presence of LINC00858. Moreover, the mRNA expression of RAN was not associated with LINC00858 in the TCGA-STAD database (Fig. 4C). The ubiquitin–proteasome system is one of the major pathways responsible for the degradation of proteins [21]. Following treatment with the proteasomal inhibitor MG132, RAN protein level was intriguingly recovered even after knockdown of LINC00858 (Fig. 4D). In addition, immunoprecipitation (IP) assays also showed a significant increase in ubiquitination levels when LINC00858 was silenced in HGC27 and MKN74 cells (Fig. 4E). Upon treatment with the protein synthesis inhibitor cycloheximide (CHX), HGC27 and MKN74 cells with LINC00858 knockdown exhibited a shorter RAN half-life than the control cells (Fig. 4F and Additional file 1: Fig. S3B), indicating that LINC00858 might inhibit the proteasome-dependent degradation of RAN in GC. In order to confirm that RAN is a downstream mediator of LINC00858, we performed rescue experiments by overexpressing RAN in LINC00858 knockdown cells. The results showed that the antimetastatic effects of LINC00858 knockdown was reversed by overexpression of RAN in both HGC27 and primary GC cells (Fig. 4G). Collectively, these results reveal that LINC00858 could protect RAN from ubiquitination and proteasomal degradation, and overexpression of RAN might mediate the prometastatic effects of LINC00858 in GC.

### The transcription factor YY1 upregulates LINC00858 expression.

**Fig. 5:**
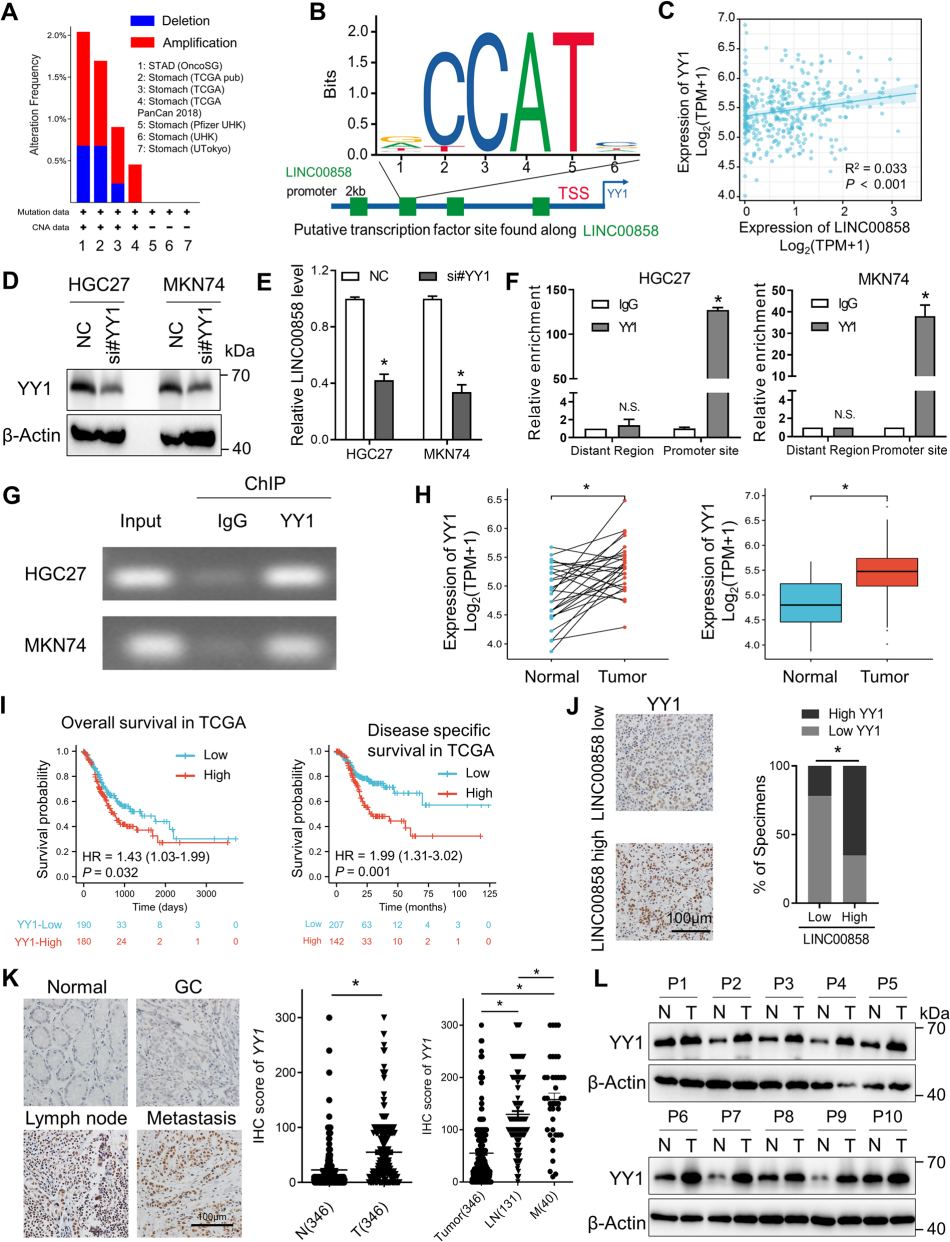
The transcription factor YY1 upregulates LINC00858 expression. **A** Genomic alteration of LINC00858 in GC was analyzed from the cBioPortal dataset. **B** The transcription factor YY1 binding motif was predicted by bioinformatic analysis. A scheme of the LINC00858 promoter with a YY1 binding site downstream of the TSS is shown. **C** Correlations between YY1 and LINC00858 in the TCGA-STAD database. **D**, **E** Western blotting of YY1 protein (**D**) and qPCR of LINC00858 (**E**) expression in HGC27 and MKN74 cells transfected with siRNAs. **F** ChIP-qPCR assay indicating the direct binding of YY1 to the LINC00858 promoter region in HGC27 and MKN74 cells. **G** Electrophoresis of the qPCR products indicates the direct binding of YY1 to the LINC00858 promoter region in HGC27 and MKN74 cells. **H** YY1 expression in paired and unpaired TCGA-STAD tumors and adjacent normal tissues. **I** Kaplan–Meier analysis of the correlation between YY1 expression and overall survival (left) and disease specific survival (right) in the TCGA-STAD database. **J** Representative images of immunohistochemical analysis of YY1 in LINC00858 low- and high-expressing samples (left panel). Associations between LINC00858 expression and protein level of YY1 in GC tissues (right panel). **K** Representative IHC images of YY1 in the adjacent normal tissues, GC, lymph nodes and distant metastasis. Scale bars = 100 μm (left panel). IHC scores of YY1 expression were calculated in the right panel. **L** Western blotting detection of YY1 expression indicates higher expression in tumor tissues than paired adjacent normal tissues in 10 patients from SYSUCC. The *P* values in E, F, H and K were calculated using a two-sided paired and unpaired Student’s t test. The *P* value in J (right) was calculated using chi-square test *P < 0.05

As described above, LINC00858 is overexpressed in GC and is associated with poor clinical outcome. However, the specific mechanism of its upregulation warrants further exploration. Through mining the cBioPortal dataset, a less than 2% amplification frequency of LINC00858 was observed in the DNA of GC samples (Fig. 5A). We therefore focused on the transcriptional regulation of LINC00858 and analyzed the 2-kb upstream and 0.5-kb downstream ranges of the LINC00858 gene in the JASPAR database (http://jaspar.genereg.net/) (Fig. 5B). The results showed that the promoter region of LINC00858 had four binding sites for the transcription factor YY1 (Fig. 5B), indicating that LINC00858 may be regulated by YY1, which was further validated by a positive correlation between LINC00858 and YY1 in TCGA-STAD database (Fig. 5C). To test this hypothesis, we transfected HGC27 and MKN74 cells with siRNAs targeting YY1 and found significant downregulation of LINC00858 upon knockdown of YY1 (Fig. 5D and E). Subsequent ChIP-qPCR assays confirmed that YY1 directly binds to the LINC00858 promoter near the transcription start site (TSS) in both HGC27 and MKN74 cells (Fig. 5F). Electrophoresis of the qPCR products also confirmed this binding, and no bands were evident in the immunoprecipitants obtained with control IgG (Fig. 5G).

Finally, YY1 expression was first detected in the TCGA-STAD database. In the paired and unpaired analysis, significant overexpression of YY1 was observed in tumor tissues compared with normal tissues (Fig. 5H) and was associated with poor overall survival and disease specific survival (Fig. 5I). Expression of YY1 was further detected via IHC in a panel of GC samples. As shown in Fig. 5J, upregulated LINC00858 was found to be positively associated with YY1 in 55 GC samples. Moreover, YY1 was significantly overexpressed in the GC compared with adjacent normal tissues. Compared with primary GC tissues, YY1 was gradually increased in lymph nodes and distant metastasis (Fig. 5K), indicating vital roles of YY1 in mGC. We then evaluate YY1 expression in our collected fresh gastric samples and found YY1 was remarkably overexpressed in tumors compared with normal counterparts (Fig. 5L), which is in accordance with previous reports [22–24] and indicates an oncogenic role of YY1 in GC. Collectively, upregulation of LINC00858 was transcriptionally regulated by YY1, which was significantly overexpressed in GC.

## Discussion

Over the past years, we have identified the lncRNAs MNX-AS1 [15] and AGPG [16] as vital regulators of the progression of colorectal cancer and esophageal squamous cell carcinoma, respectively. However, lncRNAs associated with GC metastasis remain largely unknown, while an increasing number of studies have confirmed that lncRNAs are essential for the onset and progression of GC [8, 9, 25]. The fact that the median survival of GC patients with distant metastasis versus early-stage disease is less than 1.5 year versus over 5 years accentuates the unmet clinical need of GC. We therefore focused on lncRNAs upregulated in GC and associated with poor survival, and our in vitro and in vivo assays showed that LINC00858 might play a role in the metastasis of GC.

Malignant transformation requires alteration of signaling flow and disruption of intracellular homeostasis. Numerous studies have demonstrated that lncRNAs may lie at the heart of cellular information flux, as they can act as decoys, guides or scaffolds for proteins and other noncoding RNAs [7]. Overexpression of LINC00858 has been reported to promote proliferation and migration of non-small cell lung cancer and colorectal cancer by acting as a competing endogenous RNA [26, 27]. In our study, we found that knockdown of LINC00858 suppressed migration and invasion of GC cells as well as primary cells isolated from a fresh gastric cancer sample. These oncogenic roles of LINC00858 were in concordance with previous reports [28–31]. However, none of the abovementioned studies have reported the use of in vivo GC metastatic models. We therefore, for the first time, explored the roles of LINC00858 using tail vein injection and gastric orthotopic transplantation models, hence providing in vivo evidences of the prometastatic roles of LINC00858 in GC.

As a member of the GTPase family, RAN cannot attach to subcellular membranes due to a lack of the unique anchoring CAAX motif, and it is distributed in both the nucleus and cytoplasm, which is completely different from the subcellular location patterns of other GTPases [10]. Thus, the findings that LINC00858 was mainly localized in the cytoplasm and could bind with RAN in our study were reasonable. The prometastatic roles of RAN have been reported in several tumors [32, 33] apart from its basic functions as a key regulator of bioactive molecule shuttling during cell interphase and mitosis [34]. Overexpression of RAN was associated with induction of EMT (increased N-cadherin and decreased E-cadherin expression) in non-small cell lung cancer cells through a PI3K-dependent and MAPK-independent pathway [33] and mediated the prometastatic roles of the glycophosphoprotein osteopontin in breast cancer [32]. Previous reports have shown that the RNA-binding protein LIN28B is responsible for the overexpression of RAN by directly interacting with and stabilizing the mRNA [13], while the transcription factor c-Myc could transactivate the promoter of RAN to induce its overexpression [35]. However, the posttranscriptional regulation of RAN in GC remains unknown. We showed that the ubiquitin-mediated degradation of RAN was influenced by LINC00858 in GC in this study.

Both oncogenic and tumor suppressive roles of the transcription factor YY1 via regulation of cell proliferation and tumor metastasis have been reported [36]. D. Bhaskar Rao et al. showed that knockdown of YY1 in gastric cancer cells resulted in inhibition of the Wnt/β-catenin, JNK/MAPK, ERK/MAPK, and HIF-1α signaling pathways [37], all of which have been shown to play vital roles in malignant metastasis. Our data showed that YY1 could bind to the promotor of LINC00858 and induce its expression, which was confirmed by YY1 binding to the predicted sites of the promotor region of LINC00858. We therefore speculate that YY1 may be, at least partially, responsible for the aberrant expression of LINC00858 in GC. Moreover, YY1 has been shown to regulate a series of noncoding RNAs such as lncRNAs and microRNAs, in several tumors including GC [37, 38], which was further validated in our study. As the oncogenic roles of LINC00858 have been established in GC via in vivo, in vitro and clinical analyses and YY1 has been explored as a therapeutic target [37], a significant role of the YY1/LINC00858 pathway in diagnosis, targeted therapy and combination therapy is likely, and those prior studies have set the foundation for further clinical studies.

**Fig. 6:**
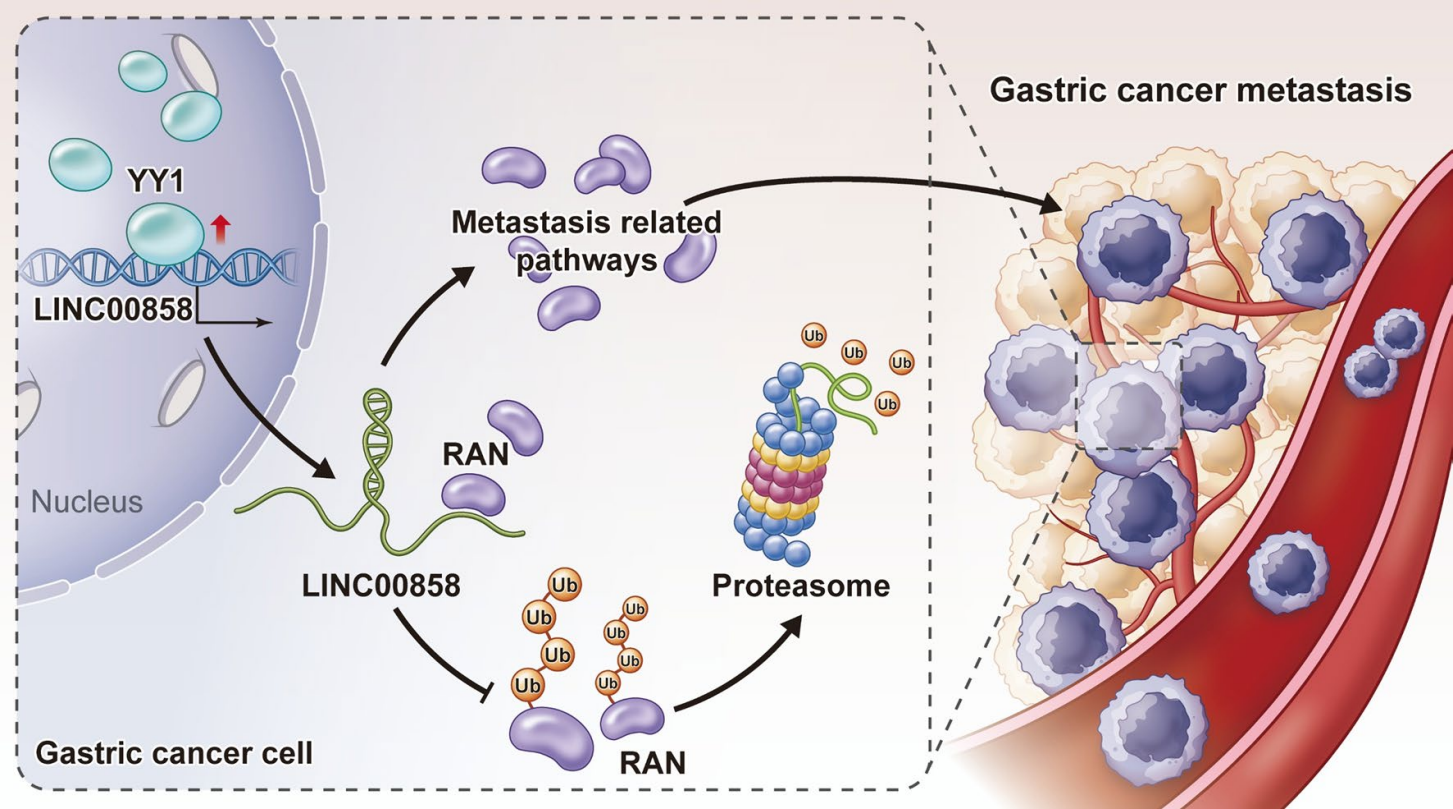
Proposed working models of this study

Our study demonstrates that the GC-associated lncRNA LINC00858 is an oncogenic lncRNA that promotes metastasis in vitro and in vivo. Mechanistically, LINC00858 could interact with RAN and thus protect it from degradation by the ubiquitin system. The transcription factor YY1 was found to bind the promoter of LINC00858 to upregulate its expression. Moreover, upregulation of LINC00858 was positively associated with expression of YY1 and RAN in GC samples (Fig. 6). Altogether, the data from our study showed the prometastatic roles of LINC00858 and elucidated its upstream and downstream effectors in GC, which might pave the way for further in-depth studies.

## Supplementary Information

**Additional file 1**. Supplementary Figures 1–3 and Tables 1–6.

## Data Availability

All data are within the main text or its Supplementary Information. The other datasets used and/or analyzed during the current study are available from the corresponding author on reasonable request.
