# Supplementary material for: LINC00858 stabilizes RAN expression and promotes metastasis of gastric cancer
Source: Biol Direct. 2022 Dec 17;17:41. doi: 10.1186/s13062-022-00355-5 (PMC9759904; doi:10.1186/s13062-022-00355-5)
Supplement: Supplementary file 1 — Additional file 1. Supplementary Figures 1–3 and Tables 1–6. [file 13062_2022_355_MOESM1_ESM.pdf]

Supplementary Figure 1

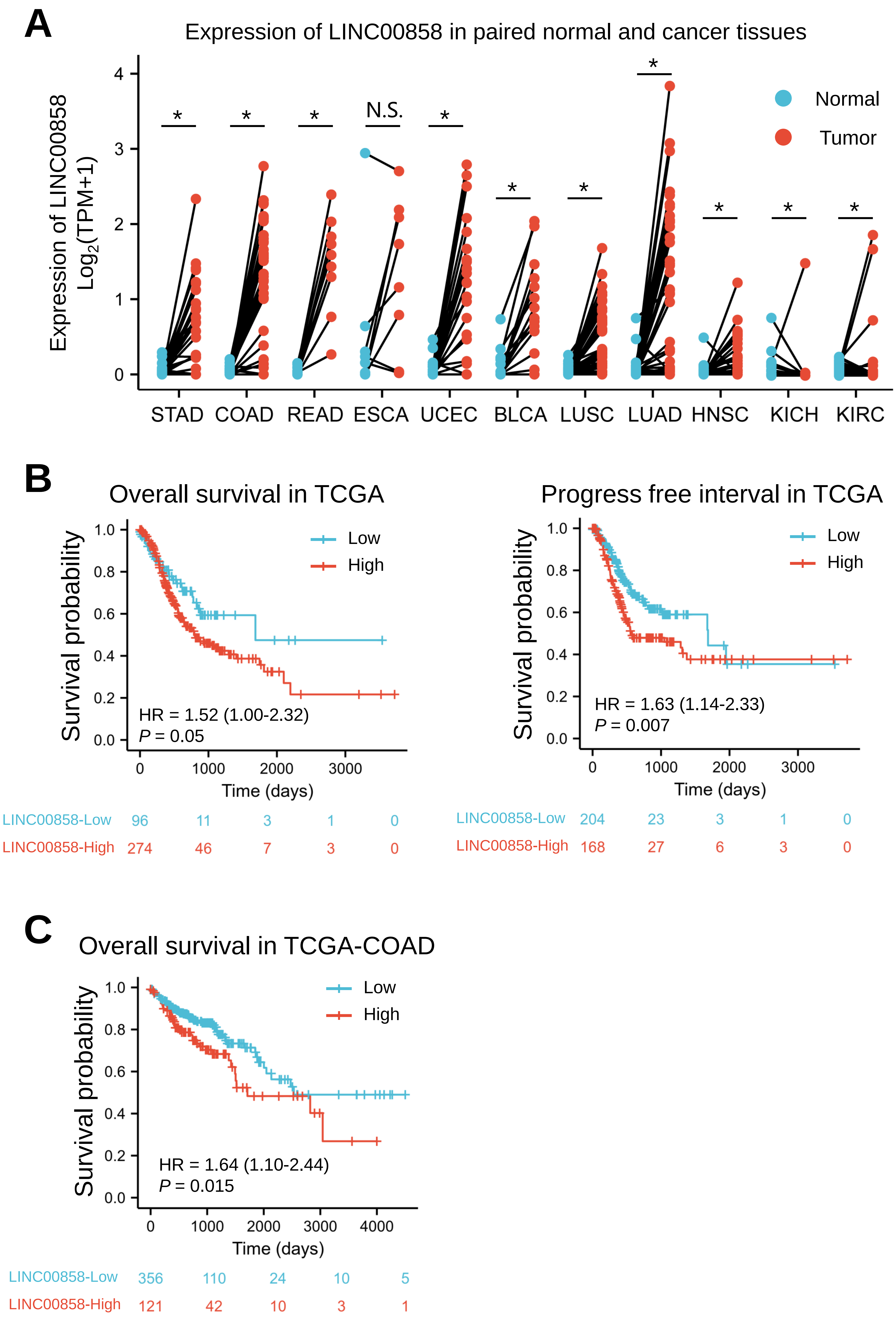

Supplementary Figure 2

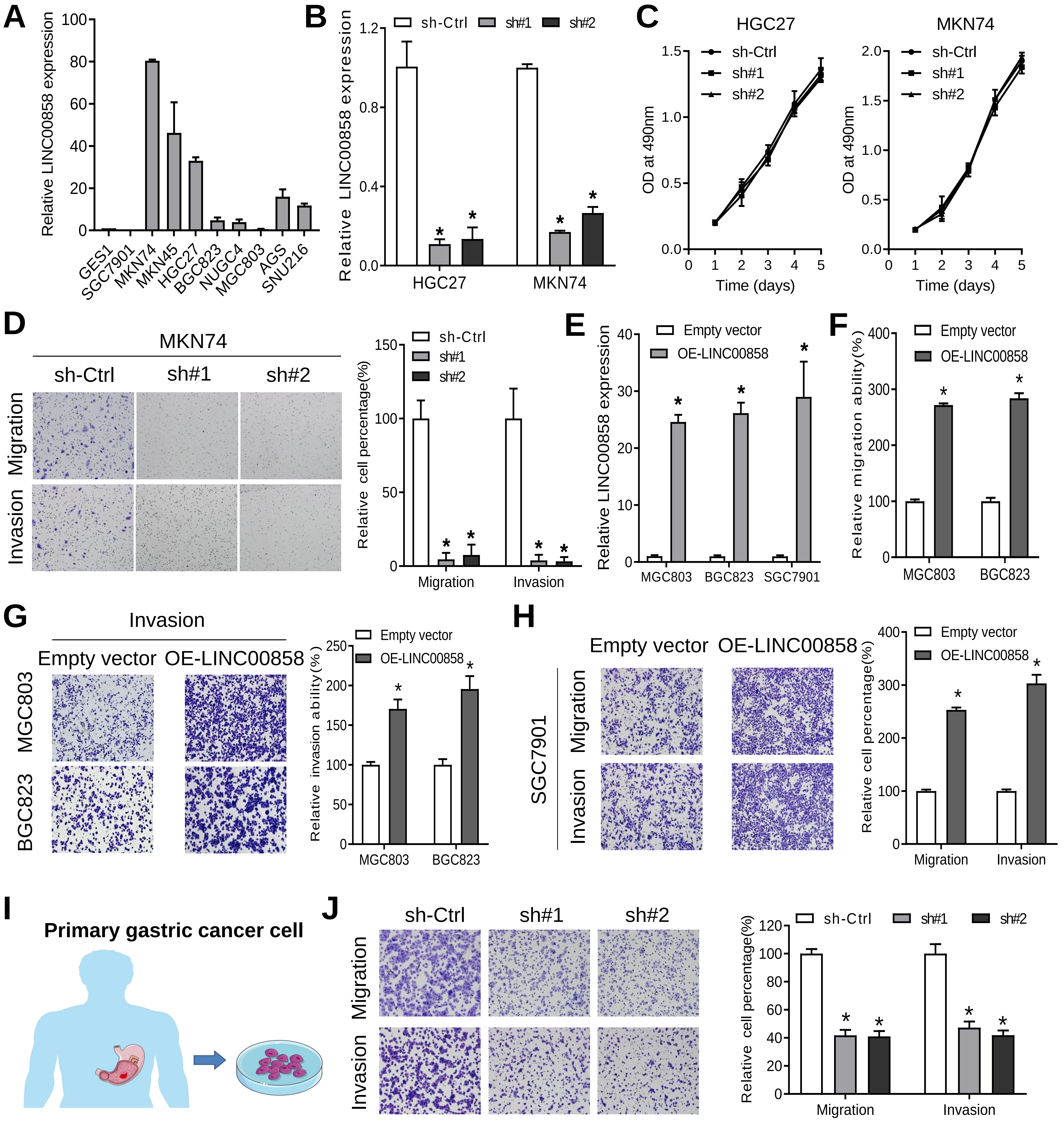

Supplementary Figure 3

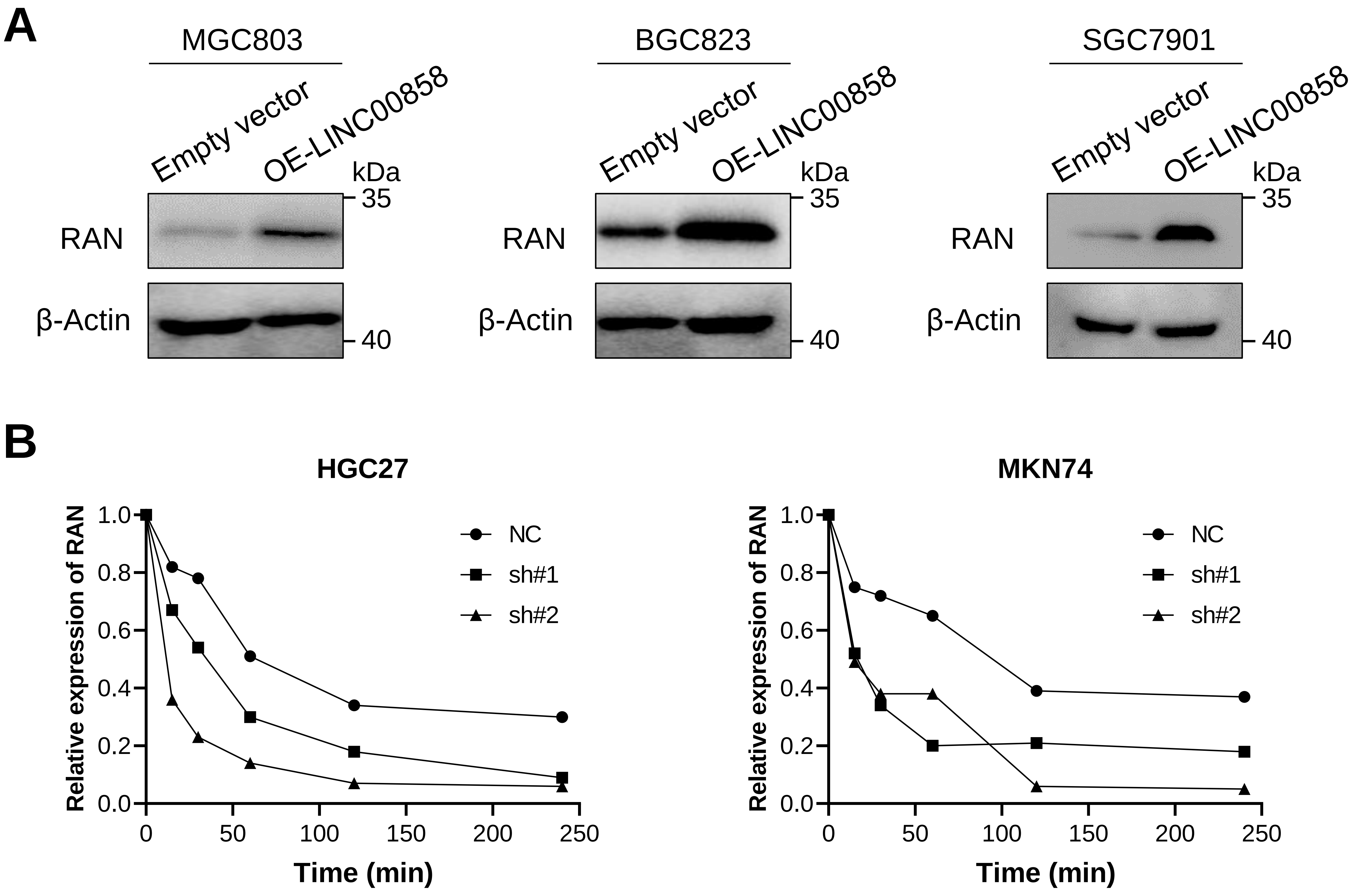

Supplementary figure legends

Figure S1. LINC00858 is overexpressed across human cancers. (A) LINC00858 expression in TCGA tumors and adjacent normal tissues. Kaplan-Meier analysis of the correlation between LINC00858 expression and overall survival (B) and progress free interval (C) in the TCGA-STAD database. (D) Kaplan-Meier analysis of the correlation between LINC00858 expression and overall survival in the TCGA-COAD database. $*P < 0.05$ and N.S. indicates nonsignificant.

Figure S2. Overexpression of LINC00858 promotes the migration and invasion of GC cells. (A) qPCR analysis of LINC00858 expression in GES1, SGC7901, MKN74, MKN45, HGC27, BGC823, NUGC4, MGC803, AGS and SNU216 cells. (B) qPCR detection of LINC00858 in MKN74 and HGC27 cells after transfection with shRNAs. (C) Cell proliferation of HGC27 and MKN74 was assessed by MTS assays (OD 490 nm). (D) Representative images (left panel) and quantification (right panel) of migration and invasion in MKN74 cells upon knockdown of LINC00858. (E) qPCR detection of LINC00858 in MGC803, BGC823 and SGC7901 cells after overexpression of LINC00858. (F) Quantification of migrated MGC803 and BGC823 cells upon overexpression of LINC00858. (G) Representative images (left panel) of invaded MGC803 and BGC823 cells upon overexpression of LINC00858. Quantification data are shown in the right panel. (H) Representative images (left panel) and quantification (right panel) of migration and invasion in SGC7901 cells upon overexpression of LINC00858. (I) Schematic diagram showing the generation of isolated primary GC cells. (J) Representative images (left panel) and quantification (right panel) of migration and invasion in primary GC cells upon knockdown of LINC00858. The *P* values in B, D, E, F, G, H and J were calculated using a two-sided unpaired Student's t test. $*P < 0.05$.

Figure S3. LINC00858 regulates expression of RAN in GC. (A) Western blotting of RAN expression in MGC803, BGC823 and SGC7901 cells upon overexpression of LINC00858. (B) Quantitative analysis of RAN expression upon knockdown of LINC00858 in HGC27 and MKN74 cells via the Bio-Rad Image Lab Software 5.2.1.

Supplementary Table 1: Correlation between clinicopathological parameters and LINC00858 expression

	LINC00858 expression		*P*
	Low, n(%)	High, n(%)	
Age			
<60	73(57.5%)	54(42.5%)	0.016*
≥60	41(40.6%)	60(59.4%)	
Gender			
Male	80(49.4%)	82(52.6%)	0.884
Female	34(51.5%)	32(48.5%)	
Tumor size			
＜ 5cm	54(56.8%)	41(43.2%)	0.107
≥5cm	60(45.1%)	73(54.9%)	
Differentiation status			

Well or Moderate	36(43.9%)	46(56.1%)	0.214
Poor and others	78(53.4%)	68(46.6%)	
T stage			
T1-T2	21(65.6%)	11(34.4%)	0.085
T3-T4	93(47.4%)	103(52.6%)	
Lymph node stage			
N0-N1	54(62.8%)	32(37.2%)	0.004*
N2-N3	60(42.3%)	82(57.7%)	
Infiltration of peritumoral tissues			
Absent	87(54.4%)	73(45.6%)	0.059
Present	27(39.7%)	41(60.3%)	
Tumor thrombus			
Absent	103(52.8%)	92(47.2%)	0.059

Present	11(33.3%)	22(66.7%)	
Peritoneal dissemination			
Absent	105(50.2%)	104(49.8%)	0.615
Present	7(41.2%)	10(58.8%)	
TNM stage			
I-II	45(63.4%)	26(36.6%)	0.010*
III-IV	69(43.9%)	88(56.1%)	

* indicates *P* value less than 0.05.

Supplementary Table 2. Univariate and multivariate analyses of various potential prognostic factors

Clinical indices	Univariate analysis			Multivariate analysis		
	HR	95%CI	*P*	HR	95%CI	*P*
**Overall survival**						
Age (<60/≥60)	1.316	0.902-1.920	0.154	-	-	-
Gender (male/female)	1.125	0.744-1.701	0.577	-	-	-
Tumor location (down/upper or middle)	0.732	0.498-1.077	0.113	-	-	-
Tumor size (≥5cm/<5cm)	1.358	0.919-2.008	0.125	-	-	-
Differentiation (poor/well, moderate)	1.518	1.004-2.296	0.048*	1.822	1.197-2.773	0.005*
T stage (T3-T4/T1-T2)	3.089	1.435-6.650	0.004*	0.940	0.399-2.215	0.888
N stage (N2-N3/N0-N1)	2.191	1.429-3.359	0.000*	0.683	0.390-1.195	0.182
Infiltration of peritumoral tissues (present/absent)	2.665	1.813-3.918	0.000*	2.010	1.349-2.995	0.001*

TNM Stage (III-IV/I-II)	3.964	2.326-6.755	0.000*	4.461	2.140-9.301	0.000*
LINC00858 expression (high/low)	3.531	2.331-5.348	0.000*	3.184	2.057-4.927	0.000*
**Disease-free survival**						
Age (<60/≥60)	0.682	0.387-1.205	0.182	-	-	-
Gender (male/female)	1.939	1.122-3.350	0.018*	2.151	1.225-3.776	0.008*
Tumor location (down/upper or middle)	0.902	0.514-1.582	0.719	-	-	-
Tumor size (≥5cm/<5cm)	1.180	0.680-2.047	0.557	-	-	-
Differentiation (poor/well, moderate)	1.187	0.672-2.097	0.554	-	-	-
T stage (T3-T4/T1-T2)	5.466	1.330-22.47	0.019*	2.632	0.596-11.63	0.202
N stage (N2-N3/N0-N1)	2.430	1.299-4.547	0.005*	0.796	0.322-1.963	0.620
Infiltration of peritumoral tissues (present/absent)	1.572	0.880-2.807	0.127	-	-	-
TNM Stage (III-IV/I-II)	3.802	1.788-8.085	0.001*	3.180	1.059-9.550	0.039*

LINC00858 expression (high/low)	3.170	1.775-5.659	0.000*	2.861	1.583-5.170	0.000*

* indicates *P* value less than 0.05.

Supplementary Table 3: Proteins interacting with LINC00858						
Accession	Score	Mass	Matches	Sequences	emPAI	Protein description
Q12849	304	53606	23 (15)	9 (5)	0.52	G-rich sequence factor 1 OS=Homo sapiens OX=9606 GN=GRSF1 PE=1 SV=3
P52597	160	45985	12 (9)	5 (4)	0.41	Heterogeneous nuclear ribonucleoprotein F OS=Homo sapiens OX=9606 GN=HNRNPF PE=1 SV=3
P31943	205	49484	16 (11)	5 (3)	0.29	Heterogeneous nuclear ribonucleoprotein H OS=Homo sapiens OX=9606 GN=HNRNPH1 PE=1 SV=4
**P62826**	**112**	**24579**	**6 (5)**	**2 (2)**	**0.29**	**GTP-binding nuclear protein Ran OS=Homo sapiens OX=9606 GN=RAN PE=1 SV=3**
P16989	189	40066	13 (4)	6 (3)	0.27	Y-box-binding protein 3 OS=Homo sapiens OX=9606 GN=YBX3 PE=1 SV=4
Q5VTE0	125	50495	11 (5)	6 (3)	0.21	Putative elongation factor 1-alpha-like 3 OS=Homo sapiens OX=9606 GN=EEF1A1P5 PE=5 SV=1
**P05089**	**28**	**34884**	**2 (1)**	**2 (1)**	**0.1**	**Arginase-1 OS=Homo sapiens OX=9606 GN=ARG1 PE=1 SV=2**
P04406	119	36201	7 (3)	3 (1)	0.09	Glyceraldehyde-3-phosphate dehydrogenase OS=Homo sapiens OX=9606 GN=GAPDH PE=1 SV=3
**Q14103**	**21**	**38581**	**2 (1)**	**2 (1)**	**0.09**	**Heterogeneous nuclear ribonucleoprotein D0 OS=Homo sapiens OX=9606 GN=HNRNPD PE=1 SV=1**
Q15366	26	38955	3 (1)	2 (1)	0.08	Poly(rC)-binding protein 2 OS=Homo sapiens OX=9606 GN=PCBP2 PE=1 SV=1
O60506	53	69788	3 (1)	2 (1)	0.05	Heterogeneous nuclear ribonucleoprotein Q OS=Homo sapiens OX=9606 GN=SYNCRIP PE=1 SV=2
Q9H0D6	39	1E+05	10 (1)	6 (1)	0.03	5'-3' exoribonuclease 2 OS=Homo sapiens OX=9606 GN=XRN2 PE=1 SV=1
Q9C0H9	25	1E+05	10 (1)	3 (1)	0.03	SRC kinase signaling inhibitor 1 OS=Homo sapiens OX=9606 GN=SRCIN1 PE=1 SV=4
Q8TF72	21	2E+05	7 (1)	3 (1)	0.01	Protein Shroom3 OS=Homo sapiens OX=9606 GN=SHROOM3 PE=1 SV=2

Supplementary Table 4: Sequences of primers, shRNAs and siRNAs

Genes	Primers-F	Primers-R
LINC00858	CTGTGAGGCTTATGTATGGG	ACTTTCCCTTCTCTCCACTT
RAN	GGTGGTACTGGAAAAACGACC	CCCAAGGTGGCTACATACTTCT
YY1	AAGAGCGGCAAGAAGAGTTAC	CAACCACTGTCTCATGGTCAATA
LINC00858-Promoter	AGAAGACGGGTGATTTCTGC	TTGACTCGGAAAGGGAACTC

Genes	sh or si RNA
sh-LINC00858#1	GGACTTCATGGTTCAGCAT
sh-LINC00858#2	GCATGGAGAATTTAAGCAA
si#YY1	CGACGACTACATTGAACAA

Supplementary Table 5: Detailed numbers and incubation times in transwell assays						
	HGC27	MKN74	Primart GC cell	MGC803	BGC823	SGC7901
Cell number ($\times 10^4$)	15	25	15	15	15	15
Incubation time (h)	24	48	16	16	16	16

Supplementary Table 6: Full names of acronyms	
Acronyms	Full name
GC	Gastric cancer
lncRNA	Long non-coding RNA
mGC	Metastatic gastric cancer
OS	Overall survival
TCGA	The Cancer Genome Atlas
TPM	Transcripts per kilobase million
YY1	Yin Yang-1
RAN	RAN, member RAS oncogene family
qPCR	Quantitative polymerase chain reaction
STAD	Stomach cancer
GTEx	Genotype-Tissue Expression
COAD	Colon cancer
GClnc1	Gastric cancer–associated lncRNA 1
WDR5	WD repeat domain 5
KAT2A	Lysine acetyltransferase 2A
GMAN	Gastric cancer metastasis associated long noncoding RNA
GMAN-AS	GMAN antisense transcript
RhoA	Ras homolog family member A
FBS	Fetal bovine serum
cDNA	Complementary DNA
ChIP	Chromatin immunoprecipitation
RIP	RNA immunoprecipitation
SD	Standard deviation
ANOVA	Analysis of variance
CRC	Coloretal cancer
siRNA	Small interfering RNA
shRNA	Short hairpin RNA
HCMDB	Human Cancer Metastasis Database
MNX-AS1	Antisense lncRNA located on chr7: q36.3 (ENSG00000243479)
AGPG	Actin Gamma 1 Pseudogene
JNK	c-Jun N-terminal kinase
MAPK	Mitogen-activated protein kinase
ERK	Extracellular signal-regulated kinase
EMT	Epithelial-mesenchymal transition
